# Heterogeneity in *Staphylococcus aureus* Bacteraemia Clinical Trials Complicates Interpretation of Findings

**DOI:** 10.1093/infdis/jiac219

**Published:** 2022-05-27

**Authors:** Heather W Dolby, Sarah A Clifford, Ian F Laurenson, Vance G Fowler, Clark D Russell

**Affiliations:** University of Edinburgh Centre for Inflammation Research, Queen’s Medical Research Institute, Edinburgh, United Kingdom; Regional Infectious Diseases Unit, Western General Hospital, Edinburgh, United Kingdom; Clinical Microbiology, Royal Infirmary of Edinburgh, Edinburgh, United Kingdom; Division of Infectious Diseases and International Health, Department of Medicine, Duke University School of Medicine, Durham, North Carolina, USA; Duke Clinical Research Institute, Durham, North Carolina, USA; University of Edinburgh Centre for Inflammation Research, Queen’s Medical Research Institute, Edinburgh, United Kingdom; Regional Infectious Diseases Unit, Western General Hospital, Edinburgh, United Kingdom; Clinical Microbiology, Royal Infirmary of Edinburgh, Edinburgh, United Kingdom

**Keywords:** bacteremia, clinical trials, *Staphylococcus aureus*

## Abstract

We systematically evaluated randomized-controlled trials (RCTs) for Staphylococcus aureus bacteremia (SAB). There was intertrial heterogeneity in cohort characteristics, including bacteremia source, complicated SAB, and comorbidities. Reporting of cohort characteristics was itself variable, including bacteremia source and illness severity. Selection bias was introduced by exclusion criteria relating to comorbidities, illness severity, infection types, and source control. Mortality was lower in RCT control arms compared with observational cohorts. Differences in outcome definitions impedes meta-analysis. These issues complicate the interpretation and application of SAB RCT results. The value of these trials should be maximized by a standardized approach to recruitment, definitions, and reporting.

## Teaser

RCTs of medical therapy for SAB contain intertrial heterogeneity in clinically important cohort characteristics. Reporting of cohort characteristics and definitions of outcomes are variable. Some exclusion criteria introduce selection biases. These issues complicate the interpretation and application of results.

*Staphylococcus aureus* bacteremia (SAB) is a complex disease associated with persistently high mortality (15%–50%) [1]. Our understanding of optimal antimicrobial treatment is constrained by limited availability of evidence from randomized-controlled trials (RCTs). Conducting and interpreting RCTs in SAB is challenging [2] due to heterogeneity of the host, pathogen, and extent of infection. Application of therapeutic findings made in clinical trial cohorts to “real-life” patient cohorts relies on representative participant recruitment to trials and detailed reporting of cohort characteristics. Furthermore, as with other diseases, we likely fail to identify patient subgroups that would differentially benefit from specific therapies [3, 4]. In SAB, specific subgroups could hypothetically differentially benefit from combination antimicrobials or modulation of host factors. There is a need to maximize the value of RCTs for SAB, to ensure applicability of findings and to differentiate true lack of efficacy from treatment effects that are subgroup dependent.

To achieve this, SAB trial participants should represent the full spectrum of real-life patient cohorts (where appropriate for the study drug); detailed and consistent participant characteristics should be collected and reported to allow hypothesis-generating analyses in sufficiently powered subgroups; and outcome reporting should permit meaningful meta-analyses. We aimed to identify current SAB RCTs and systematically analyze the characteristics of included patients, eligibility criteria, and reported outcomes.

## METHODS

### Identification of Trials

Randomized-controlled trials of medical therapy for SAB were identified by a systematic literature review (PubMed/MEDLINE search strategy shown in Supplementary Table 1). The review protocol was prospectively registered on the PROSPERO international prospective register of systematic reviews (CRD42021262395). H.W.D. and C.D.R. independently screened search results (Supplementary Figure 1) to identify eligible studies (Supplementary Table 2). We included RCTs where all participants had confirmed bacteremia with methicillin-susceptible *S aureus* (MSSA) or methicillin-resistant *S aureus* (MRSA), and we excluded studies restricted to people with SAB from specific sources (ie, not nonselected SAB).

### Analysis

Clinical characteristics of the control arm, eligibility criteria and outcome measures were recorded. To compare cohort characteristics between trials, variables reported in >50% of trials were included and z-scores were calculated for each study ($z=\frac{\left(\mathrm{value}\;\mathrm{for}\;\mathrm{study}-\mathrm{mean}\;\mathrm{for}\;\mathrm{variable}\right)}{\mathrm{standard}\;\mathrm{deviation}\;\mathrm{for}\;\mathrm{variable}}$) and visualized in heatmaps (GraphPad Prism, v9.2.0). Trials were clustered based on cohort characteristics using network analysis and the Markov Clustering Algorithm (Graphia, v2.0) [5]. For clustering, variables with >40% missing data were excluded and missing values for the remaining variables were imputed using chained random forests with 500 iterations and predictive mean matching *k* = 5 (*missRanger* package; RStudio, v1.3.959) [6]. Exclusion criteria were classified as strongly/potentially/poorly justified according to the framework described by Van Spall et al [7]. Justification was assessed independently by 2 authors and adjudicated by a third. Data distributions were assessed by the Shapiro-Wilk test then compared by *t* test or Mann-Whitney test as appropriate.

## RESULTS

### Identified Trials

**Figure 1.**
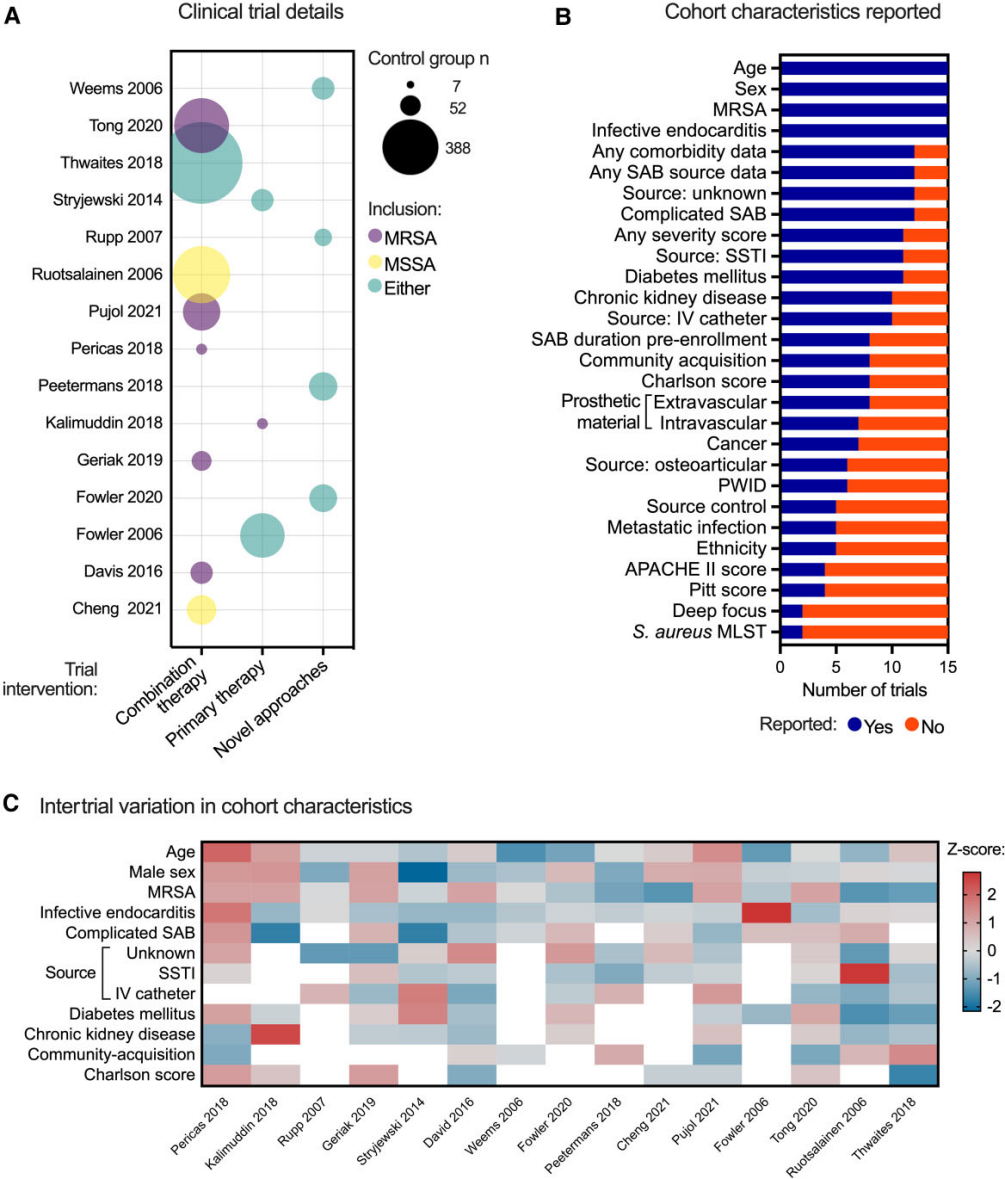
Trial and cohort characteristics of 15 *Staphylococcus aureus* bacteremia (SAB) randomized-controlled trials (RCTs). *A*, Details of SAB RCTs. The size of the bubble is proportional to the number of participants in the control arm. Bubbles are colored according to the inclusion of methicillin-susceptible *S aureus* (MSSA), methicillin-resistant *S aureus* (MRSA), or both. *B*, Cohort characteristics reported in included trials. *C*, Variability in cohort characteristics between trials. Cells in the heatmap are shaded by z-score. Only variables reported in >50% trials were included. Blank cells represent missing values. APACHE, Acute Physiologic Assessment and Chronic Health Evaluation; IV, intravenous; MLST, multilocus sequence type; PWID, person who injects drugs; SSTI, skin and soft tissue infection.

Fifteen eligible trials were identified and included in our analysis, including a total of 2537 people (Supplementary Table 3, Supplementary Figure 2). Most trials (8 of 15) investigated combination therapy. Four investigated novel approaches (anti-*S aureus* antibodies, anti-staphylococcal lysin, or direct thrombin inhibitors) and 3 investigated primary antimicrobial regimes (Figure 1A). Six trials exclusively recruited MRSA bacteremia. Seven trials were registrational (informing regulatory approval [2]) and 8 were strategy trials (informing clinical practice), including the largest trials (Supplementary Figure 3A). Strategy trials were more likely to recruit specifically MRSA bacteremia. Most trials were conducted in the United States or Europe. Asian sites were included in 5 trials but none were conducted exclusively in Asia. No trials included African sites.

### Characteristics of *Staphylococcus aureus* Bacteremia Clinical Trial Populations

Age, sex, methicillin susceptibility, and presence of infective endocarditis (IE) were reported in all trials, but there was substantial variability in reporting of other clinically important cohort characteristics (Figure 1B). Although 12 of 15 studies reported data on the primary source of bacteremia, the potential sources that were specified were different (unknown source, 12 of 12; skin and soft tissue infection [SSTI], 11 of 12; intravascular [IV] catheter, 10 of 12; osteoarticular infection, 6 of 12). The presence of metastatic infection (5 of 15) and any deep focus (2 of 15) were infrequently reported, as was the proportion of participants undergoing source control (5 of 15). Although results of any illness severity score were reported in most trials (11 of 15), the specific scores reported varied (Pitt bacteremia score, 4 of 11; Acute Physiologic Assessment and Chronic Health Evaluation [APACHE] II, 4 of 11; Systemic Inflammatory Response Syndrome [SIRS], 2 of 11; Sequential Organ Failure Assessment [SOFA], 1 of 11). Participant ethnicity was reported in 5 of 15 trials. Strategy trials reported more cohort characteristics than registrational trials (Supplementary Figure 3B).

Intertrial heterogeneity in cohort characteristics was apparent for clinically important variables (Figure 1C and Supplementary Figure 3) including community-acquisition (range, 4.9%–62%), unknown source (0%–34%), IV catheter-related SAB (7%–56%), complicated bacteremia (0%–100%), diabetes mellitus (28%–63%), and median Charlson comorbidity index score (2–6). Skin and soft tissue infection as the source was more consistent but with 1 outlier cohort (11%–35%; outlier 69%) with a similar pattern for IE (0%–20%; outliers 57.1 and 79.1%). Although reported in <50% of trials, variation was present in the proportion of people with intravascular (reported in 7 of 15) or extravascular (8 of 15) prosthetic material: 0%–71.4% and 0–25.2%, respectively. Intertrial variation was still apparent when trials were categorized as strategy or registrational, with increased age and complicated SAB in strategy trial cohorts (Supplementary Figure 3C and D). Bacterial genotyping was conducted in 2 of 15 trials; both were MRSA strategy trials. Although ST22 and ST93 were most prevalent in both, the incidence of ST239 was different and the sequence type was often reported as “Other” (19% and 46%) (Supplementary Figure 4).

Unsupervised clustering was undertaken as an additional method to evaluate heterogeneity in cohort characteristics, identifying groups of trials with differences in the proportion of both MRSA and complicated bacteremia (Supplementary Figure 5A). When trials were then analyzed in two groups, separated based on the recruitment of exclusively MRSA bacteremia, clusters differing in (1) proportions of complicated bacteremia and chronic kidney disease emerged for MRSA-only trials (Supplementary Figure 5B) and (2) the proportion of IV catheter-related SAB for other trials (Supplementary Figure 5C).

To allow comparison of trial populations with real-life cohorts, we analyzed 17 large observational cohorts (each reporting >200 people with nonselected SAB) from 14 studies reporting on a total of 22 009 people (Supplementary Table 4). Overall, a median of 55.5% (interquartile range [IQR], 11–100) of RCT control arm participants had MRSA bacteremia, compared with 21.7% (14.1–38.3; *P* = 0.009) in observational studies. When trials including MRSA or MSSA only were excluded, there was no statistically significant difference (38.3% MRSA [IQR, 11.0–55.5] vs 21.7% [IQR, 14.1–38.3]; *P* = 0.2) (Supplementary Figure 6). Median proportions of people with IE and SAB of SSTI or unknown source were similar between trial and observational populations. The proportion of IV catheter-related SAB had a bimodal distribution among trial populations and was overall less common compared with observational studies (median 18.5% [IQR, 11–42] vs 27.5% [IQR, 24.0–36.2]; *P* = 0.03). Complicated SAB appeared to be enriched in trials (median 70.8% [IQR, 34.4–82] vs 37.6% [IQR, 35.0–40.1]; *P* = 0.04), although observational studies reported this characteristic infrequently. Compared to observational studies, the median proportion of people with diabetes mellitus was higher in trials (median 42.8% [IQR, 36.5–56] vs 29.7% [IQR, 24.4–37.5]; *P* = 0.001).

### Justification of Exclusion Criteria

**Figure 2.**
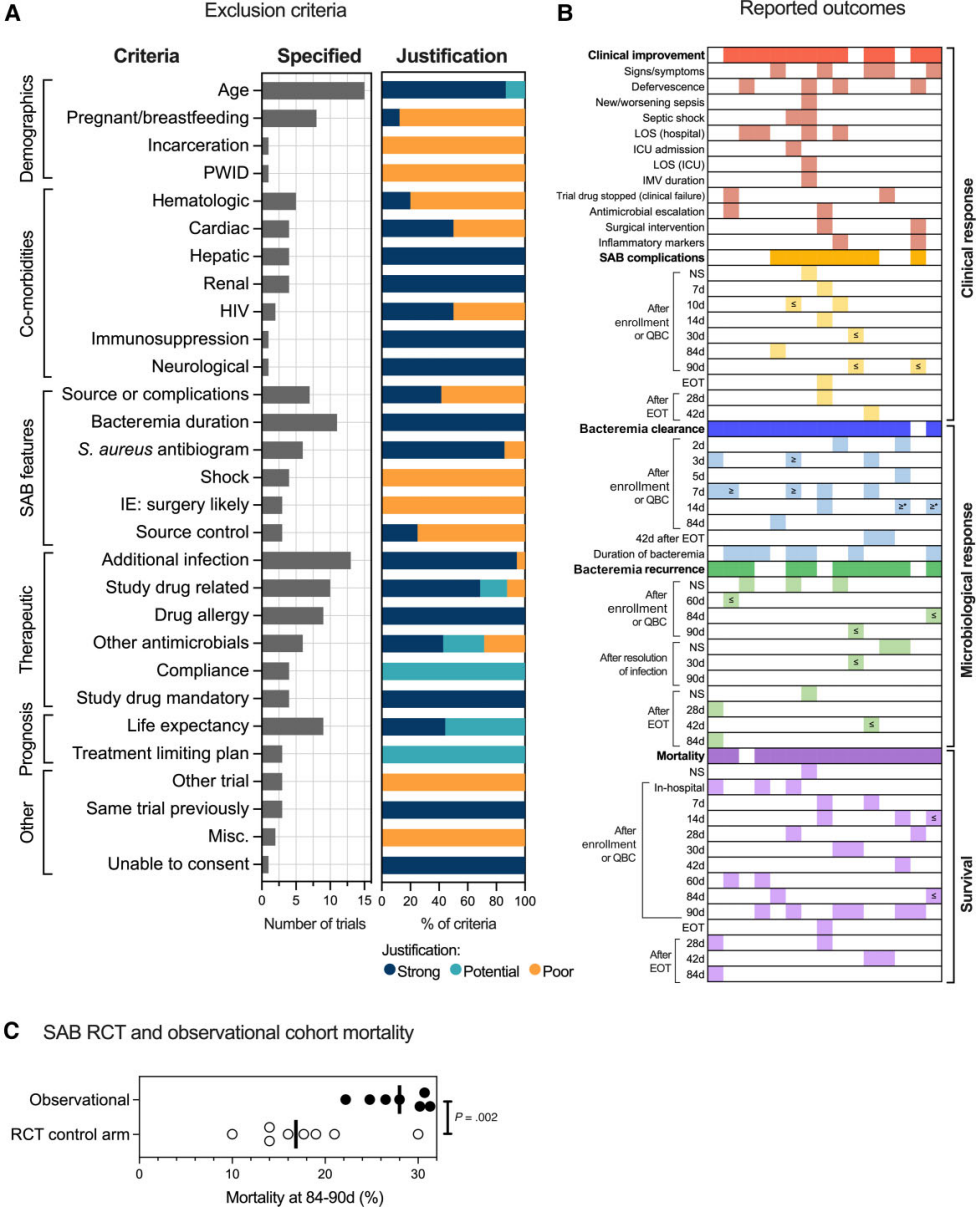
Eligibility criteria and outcomes of 15 *Staphylococcus aureus* bacteremia (SAB) randomized-controlled trials (RCTs). *A*, Reporting and justification of exclusion criteria. For each specific exclusion criteria subcategory, the number of trials specifying criteria in this subcategory is shown by the gray bar, and the justification of all criteria (as a % across all relevant trials) in the subcategory is shown in the stacked bar (shaded by classification of justification). One trial may have contributed multiple criteria to the same subcategory (see Supplementary Data File). *B*, Reporting and comparability of outcomes. Shaded cells in the grid represent an outcome (row) reported by a trial (column). If a cell is blank, the outcome was not reported in the trial. Different colors represent different outcome categories. Symbols used in grid: ≥ at/after timepoint;  ≤ up to/at timepoint; * sterile site sample (ie, not exclusively blood culture). Enrollment refers to randomization or start of trial drug. *C*, Comparison of 84- to 90-day mortality for control arm participants in SAB RCTs with observational cohorts. Details of included studies in Supplementary Table 4. Line shows the median. Groups compared by unpaired *t* test. d, days; EOT, end of treatment; HIV, human immunodeficiency virus; ICU, intensive care unit; IE, infective endocarditis; IMV, invasive mechanical ventilation; LOS, length of stay; Misc., miscellaneous; NS, not specified; PWID, person who injects drugs; QBC, qualifying blood culture.

Assessment of exclusion criteria allows more granular assessment of the external validity of a trial population than reported cohort characteristics alone. Age (to identify adults), presence of additional infections requiring antimicrobials, and bacteremia duration pre-enrollment were the most common exclusion criteria (Figure 2A). “Additional infections” mostly consisted of exclusion of polymicrobial bacteremias or coinfections requiring additional antimicrobials, and 17 of 18 were considered strongly justified on the basis that the requirement for potentially effective nonstudy antimicrobials represented a cointervention that could confound the trial results. Eleven trials specified limits to the duration of SAB before randomization in keeping with US Food and Drug Administration requirements. Time to randomization from the initial qualifying blood culture (QBC) was within 72 hours in 7 studies, 48 hours in 2 studies, and 36 hours or 7 days in 1 study each. Exclusions based on life expectancy and nonsusceptibility to the study drug were considered justified. Some exclusion criteria were judged to be poorly justified and at risk of diminishing external validity. These included (Supplementary Table 5) excluding people with prosthetic heart valves, IE likely to undergo surgery, neutropenia, people who inject drugs, and specific SAB sources (osteomyelitis, central line) or features/severity (metastatic infection, persistent bacteremia, shock). Three studies made exclusions based on source control. Two were judged poorly justified, requiring source control within 1 day or 3 days of randomization, which is difficult to achieve in practice, whereas 1 trial required intervention within 4 days, which has previously been considered practical and thus justified [2].

### Reporting and Comparability of Outcomes

Most studies reported outcomes relating to clinical response (clinical improvement or SAB complications, 13 of 15 trials), microbiological response (bacteremia clearance or recurrence, 14 of 15), and survival (14 of 15). However, substantial differences existed in the specific definitions and timepoints used (Figure 2B). Duration of bacteremia was reported as a continuous variable in 7 of 14 trials. Other trials reported bacteremia clearance at specific timepoints: between 2 and 84 days after enrollment/QBC, or 42 days after end of treatment. Differences in the definition of “clearance” were also present (Supplementary Table 6). Similar variability was present in the assessment of bacteremia recurrence (Figure 2B) and microbiological definitions (Supplementary Table 6). The most common mortality-related timepoint was 90 days after enrollment/QBC (n = 6). To permit comparison of mortality between RCT control arm and observational cohorts, we selected studies reporting mortality at 84–90 days (8 RCTs, 6 observational studies) (Supplementary Table 7). Control arm mortality was substantially lower in RCTs compared with mortality observed in real-life cohorts: mean 17.7% (standard deviation [SD] ± 6) vs 27.7% (SD ± 3.4); *P* = 0.002 (Figure 2C).

## DISCUSSION

We have used data-driven approaches to systematically evaluate current RCTs of medical therapy for SAB. Most trials investigated combination therapy, and strategy trials focused on MRSA bacteremia. Intertrial heterogeneity in key cohort characteristics is apparent. The variability in collecting and reporting of cohort characteristics, including source of bacteremia, community acquisition, and presence of metastatic infection or deep foci impedes interpretation of trial results and exploration of subgroup-dependent treatment effects. These findings are of particular importance when considering the association of noneradicated foci [8], community-acquired SAB [9], and SAB of unknown source [1] with mortality, and the possible subgroup effect in people with a deep focus seen in the ARREST trial [10]. Bacterial genotyping was undertaken in 2 of 15 trials. Bacterial genetic variation is associated with different SAB clinical phenotypes [11] and could contribute to differential treatment responses, especially to novel therapies (eg, monoclonal antibodies or lytic agents) in which conventional antimicrobial susceptibility testing is not applicable. Investigation of this could contribute to patient stratification and is important considering the changes in prevalent *S aureus* clones over time [11]. Although most exclusion criteria were considered justified, some relating to patient comorbidities, illness severity, SAB features, and timing of source control introduce potentially problematic selection biases. A likely consequence of these issues is the substantially lower mortality in RCT control arm cohorts compared with real-life observational cohorts. Overall, variation in RCT cohort characteristics and their reporting, and more subtle differences in eligibility, complicates the application of trial results. In addition, intertrial incompatibility in the definitions of microbiologic and mortality outcomes precludes useful meta-analyses. A limitation of our analysis is the potential for geographic variation in characteristics and the change in treatment patterns over time, both of which could influence outcomes [12]. Future work could apply the approach taken here to RCTs investigating other infections.

## CONCLUSIONS

*Staphylococcus aureus* bacteremia is a heterogeneous disease, but we contend this is a feature that should be exploited. Components of host and pathogen heterogeneity in SAB are likely to be amenable to different therapeutic strategies. Clinical data from SAB RCTs could contribute to identification of therapeutically relevant patient subgroups, requiring standardized collection of cohort characteristics, consistent definition of outcomes, stratification of enrollment according to SAB features (eg, source) [2], and agreement on exclusion criteria that are practical but do not diminish applicability of results. Implementation will be challenging, but the research response to the coronavirus disease 2019 pandemic illustrates that globally collaborative observational research and standardized data collection are feasible: the ISARIC (International Severe Acute Respiratory and emerging Infections Consortium) global dataset currently includes 800 459 patients from 1701 institutions in 60 countries [13]. All data were recorded using the same case report form, developed through an international consensus-building process [13, 14]. Combined with the spirit of open science, this approach could be applied to RCT conduct and add substantial value to future SAB RCTs.

## Supplementary Material

jiac219_Supplementary_Data

## Supplementary Data

Supplementary materials are available at *The Journal of Infectious Diseases* online (http://jid.oxfordjournals.org/). Supplementary materials consist of data provided by the author that are published to benefit the reader. The posted materials are not copyedited. The contents of all supplementary data are the sole responsibility of the authors. Questions or messages regarding errors should be addressed to the author.

## Notes

***Acknowledgments.*** We gratefully acknowledge Professors Steven Tong and George Sakoulas for sharing data.

***Financial support.*** This work was funded in part by the following grants: Edinburgh Clinical Academic Track (ECAT)/Wellcome Trust PhD Training Fellowship for Clinicians award 214178/Z/18/Z (to C. D. R.) and R01-AI165671 (to V. G. F.). Funding to pay the Open Access publication charges for this article was provided by the University of Edinburgh.

***Potential conflicts of interest.*** V. G .F. reports personal fees from Novartis, Debiopharm, Genentech, Achaogen, Affinium, Medicines Co., Bayer, Theravance, Affinergy, Janssen, Contrafect, Regeneron, Basilea, Destiny, Amphliphi Biosciences, Armata, Integrated Biotherapeutics, C3J; grants from National Institutes of Health, MedImmune, Cerexa/Forest/Actavis/Allergan, Pfizer, Advanced Liquid Logics, Theravance, Novartis, Cubist/Merck, Medical Biosurfaces, Locus, Affinergy, Contrafect, Karius, Genentech, Regeneron, Basilea, Janssen; Stock options from Valanbio and ArcBio; Royalties from UpToDate; honoraria from Infectious Diseases Society of America for Associate Editor Role on *Clinical Infectious Diseases*; and a patent pending in sepsis diagnostics.
