# Supplementary material for: Heterogeneity in Staphylococcus aureus Bacteraemia Clinical Trials Complicates Interpretation of Findings
Source: J Infect Dis. 2022 May 27;226(4):723–8. doi: 10.1093/infdis/jiac219 (PMC9441204; doi:10.1093/infdis/jiac219)
Supplement: jiac219_Supplementary_Data [file jiac219_supplementary_data.pdf]

Heterogeneity In *Staphylococcus aureus* Bacteraemia Clinical Trials Complicates Interpretation Of Findings

Supplementary Material

Contents:

Supplementary Table 1: Search key words 2
Supplementary Table 2: Criteria used to identify RCTs of medical therapy for non-selected SAB 3
Supplementary Table 3: Summary of 15 included SAB RCTs 4
Supplementary Table 4: Included observational cohort studies (n=14) 5
Supplementary Table 5: Clinically-relevant poorly justified exclusion criteria 6
Supplementary Table 6: Microbiologic outcome definitions 7
Supplementary Table 7: SAB RCT and observational study 84-90d mortality 8
Supplementary Figure 1: Flow diagram of study identification 9
Supplementary Figure 2: Cumulative recruitment to SAB RCTs 10
Supplementary Figure 3: Comparison of registrational and strategy trials 11
Supplementary Figure 4: Variation in *S. aureus* sequence types between trials 12
Supplementary Figure 5: Network analysis of SAB RCT cohort characteristics 13
Supplementary Figure 6: SAB RCT and observational study cohort characteristics 14
Supplementary references 15

Supplementary Table 1: Search key words

((“staphylococcus aureus” OR “s. aureus” OR “MRSA” OR “aureus” OR “MSSA” OR “staphylococc*”) AND (“bacteraemia” OR “bloodstream” OR “bacteremia” OR “BSI” OR “blood” OR “sepsis” OR “septicemia” OR “septicaemia” OR “intravascular”)) AND “trial”.

No date restriction on search
Search performed on 12/07/2021

Supplementary Table 2: Criteria used to identify RCTs of medical therapy for non-selected SAB

Inclusion criteria	Exclusion criteria
• Blood culture confirmed SAB<br>• Included MSSA, MRSA or both<br>• RCT of medical therapy (including conventional antimicrobials or novel therapies)<br>• Any phase of trial<br>• Recruited adults (≥16 years)<br>• Recruited hospitalised patients	• Not English language<br>• No control arm (usual care or placebo)<br>• Observational studies<br>• PK/PD studies<br>• Secondary analyses of other trials<br>• No full text available<br>• Included polymicrobial bacteraemias<br>• Included *S. aureus* infections without blood culture confirmed bacteraemia<br>• Exclusively recruited SAB from one source (e.g., only IV catheter-related bacteraemia or infective endocarditis)

PK: pharmacokinetic; PD: pharmacodynamic

Supplementary Table 3: Summary of 15 included SAB RCTs

Trial	Recruitment location(s)	Funding	Category	Purpose	Intervention	N screened	N included	N control*	N intervention*	Completed as planned
Fowler *et al*, 2006 [1]	USA, Belgium, Lebanon, Germany	Industry-sponsored	Primary therapy	Registrational	Daptomycin vs. SOC	–	246	115	120	Yes
Ruotsalainen *et al*, 2006 [2]	Finland	Investigator-initiated	Combination therapy	Strategy	Adjunctive levofloxacin	1226	381	190	191	Yes
Weems *et al*, 2006[3]	USA	Industry-sponsored	Novel approach	Registrational	Adjunctive tefibazumab	–	63	30	30	Yes
Rupp *et al*, 2007 [4]	USA	Industry-sponsored	Novel approach	Registrational	Adjunctive Altastaph	–	40	18	21	Yes
Stryjewski *et al*, 2014 [5]	USA, Argentina, Spain, Singapore, Hong Kong	Industry-sponsored	Primary therapy	Registrational	Telavancin vs. SOC	–	60	29	29	Yes
Davis *et al*, 2016 [6]	Australia	Investigator-initiated	Combination therapy	Strategy	Vancomycin ± flucloxacillin (MRSA)	380	60	29	31	Yes
Kalimuddin *et al*, 2018 [7]	Singapore	Investigator-initiated	Primary therapy	Registrational	Daptomycin vs. vancomycin (MRSA)	170	14	7	7	No (recruitment)
Pericas *et al*, 2018 [8]	Spain	Investigator-initiated	Combination therapy	Strategy	Fosfomycin + imipenem vs. vancomycin (MRSA)	201	15	7	8	No (recruitment)
Thwaites *et al*, 2018 [9]	UK	Investigator-initiated	Combination therapy	Strategy	Adjunctive rifampicin	2896	770	388	370	Yes
Peetermans *et al*, 2018 [10]	Belgium	Investigator-initiated	Novel approach	Registrational	Adjunctive direct thrombin inhibitor	354	94	47	47	Yes
Geriak *et al*, 2019 [11]	USA	Investigator-initiated	Combination therapy	Strategy	Daptomycin + ceftaroline vs. SOC	–	40	23	17	No (efficacy)
Tong *et al*, 2020 [12]	Australia, Singapore, Israel, New Zealand	Investigator-initiated	Combination therapy	Strategy	Vancomycin/daptomycin ± ASBL (MRSA)	1431	356	175	170	No (safety)
Fowler *et al*, 2020 [13]	USA, Belgium, Bulgaria, Chile, France, Germany, Greece, Guatemala, Israel, Italy, Russia, Spain, UK	Industry-sponsored	Novel approach	Registrational	Adjunctive Exebecase	3729	116	45	71	Yes
Pujol *et al*, 2021 [14]	Spain	Investigator-initiated	Combination therapy	Strategy	Daptomycin ± Fosfomycin (MRSA)	674	167	81	74	Yes
Cheng *et al*, 2021 [15]	Canada	Investigator-initiated	Combination therapy	Strategy	Adjunctive daptomycin	331	115	51	53	Yes

SOC: standard of care; ASBL: anti-staphylococcal beta-lactam
*eligible and included in analysis

Supplementary Table 4: Included observational cohort studies (n=14)

Study	Location	N included
Fowler *et al*, 2003 [16]	USA	724
Kaasch *et al*, 2014 [17]	Germany	912 (“INSTINCT”)
	Spain	527 (“ES1”)
	UK	1459 (“UKCIRG”)
	USA	329 (“SABG”)
Kaech *et al*, 2006 [18]	Switzerland	308
Laupland *et al*, 2008 [19]	Canada	1542
Le Moing *et al*, 2015 [20]	France	2008
Turnidge *et al*, 2009 [21]	Australia	1994
Morris & Russell, 2016 [22]	UK	556
Tong *et al*, 2012 [23]	Australia	7539
Jenkins *et al*, 2008 [24]	USA	234
Robinson *et al,* 2012 [25]	Australia	599
Fowler *et al*, 1998 [26]	USA	244
Khatib *et al*, 2006 [27]	USA	245
Willekens *et al*, 2021 [28]	Spain	441
Soulie *et al*, 2019 [29]	USA	2348

Supplementary Table 5: Clinically-relevant poorly justified exclusion criteria

Exclusion	N trials
SAB source	
Osteomyelitis	1
Central line	1
SAB features/severity	
Metastatic infection	1
Persistent bacteraemia	1
Shock	4
IE likely to undergo surgery	3
Patient characteristics	
Prosthetic heart valves	1
Neutropenia	3
Person who injects drugs*	1
Source control	
Treatable source will not removed/debrided within 72h of randomisation	1
Removable source of infection not planned to be removed within 24h	1

*Based on the Van Spall framework[30], exclusion can be *potentially* justified on the basis that an individual "may not adhere to intervention" or "may not complete follow-up" and this could potentially be applied to people who inject drugs (PWID). For example, a recent cohort study found that of 307 PWID being treated in hospital with intravenous antimicrobials for an invasive infection, 48.8% completed IV therapy[31]. However, the framework also includes exclusion based on "Chronic health condition" as a *poorly* justified reason for exclusion. Considering the chronic nature of opioid use disorder and the relevance of injection drug use to SAB (Supplementary Figure 6) we feel that on balance exclusion of PWID is poorly justified.

Supplementary Table 6: Microbiologic outcome definitions

Outcome	Definition used	N trials
**Clearance**	*Absence of* clearance: persistently positive blood cultures at the defined timepoint	8/14
	Negative blood cultures, obtained on one day at/from the defined timepoint	3/14
	Negative blood cultures, obtained on two consecutive days at/from the defined timepoint	3/14
**Recurrence**	Positive blood culture following:	
	Clinical improvement	2/9*
	Two negative blood cultures	2/9*
	≥48 hours after ≥1 negative blood culture	2/9*
	Completion of treatment and ≥1 negative blood culture	2/9*
	≥72h after a negative blood culture	1/9*

*9/11 studies reporting recurrence provided a definition

Supplementary Table 7: SAB RCT and observational study 84-90d mortality

Study	Mortality timepoint (d)[a]	Mortality (%)[b]
**RCT**		
Geriak *et al*, 2019	90	30
Davis *et al*, 2016	90	21
Peetermans *et al*, 2018	90	19
Cheng *et al*, 2021	90	17.7
Tong *et al*, 2020	90	16
Ruotsalainen *et al*, 2006	90	14
Stryjewski *et al*, 2014	84	10
Thwaites *et al*, 2018	84	14
**Observational**		
INSTINCT	90	30.7
ES1	90	24.8
UKCIRG	90	30.2
SABG	90	22.2
Le Moing *et al*, 2015	84	31.3
Fowler *et al*, 2003	84	28[c]
Souli et al, 2019	90	26.5

Unless otherwise stated, mortality is crude/all-cause.
[a] After enrolment or qualifying blood culture
[b] Control arm for RCTs
[c] Attributable mortality

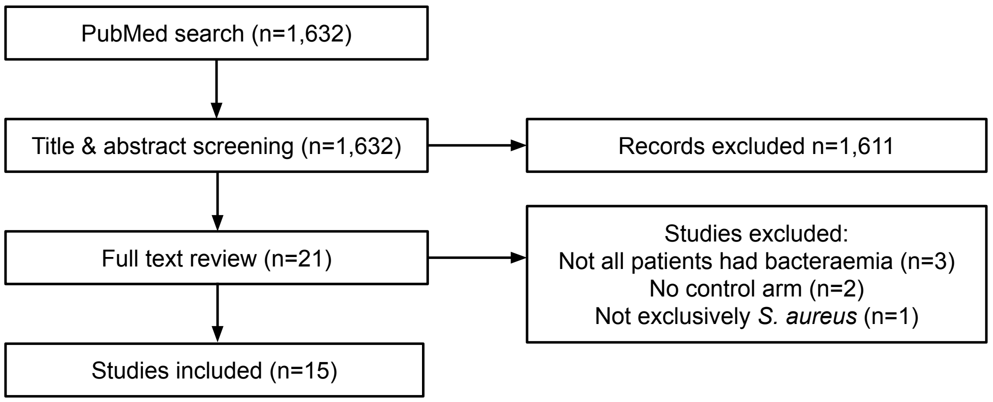

Supplementary Figure 1: Flow diagram of study identification

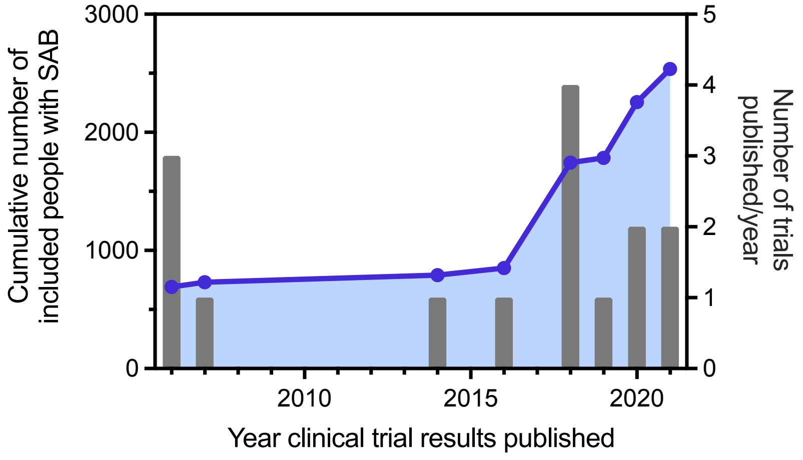

Supplementary Figure 2: Cumulative recruitment to SAB RCTs
The cumulative number of recruited people over time is shown by the blue line (left y-axis). Grey bars represent the number of trials published per year (right y-axis).

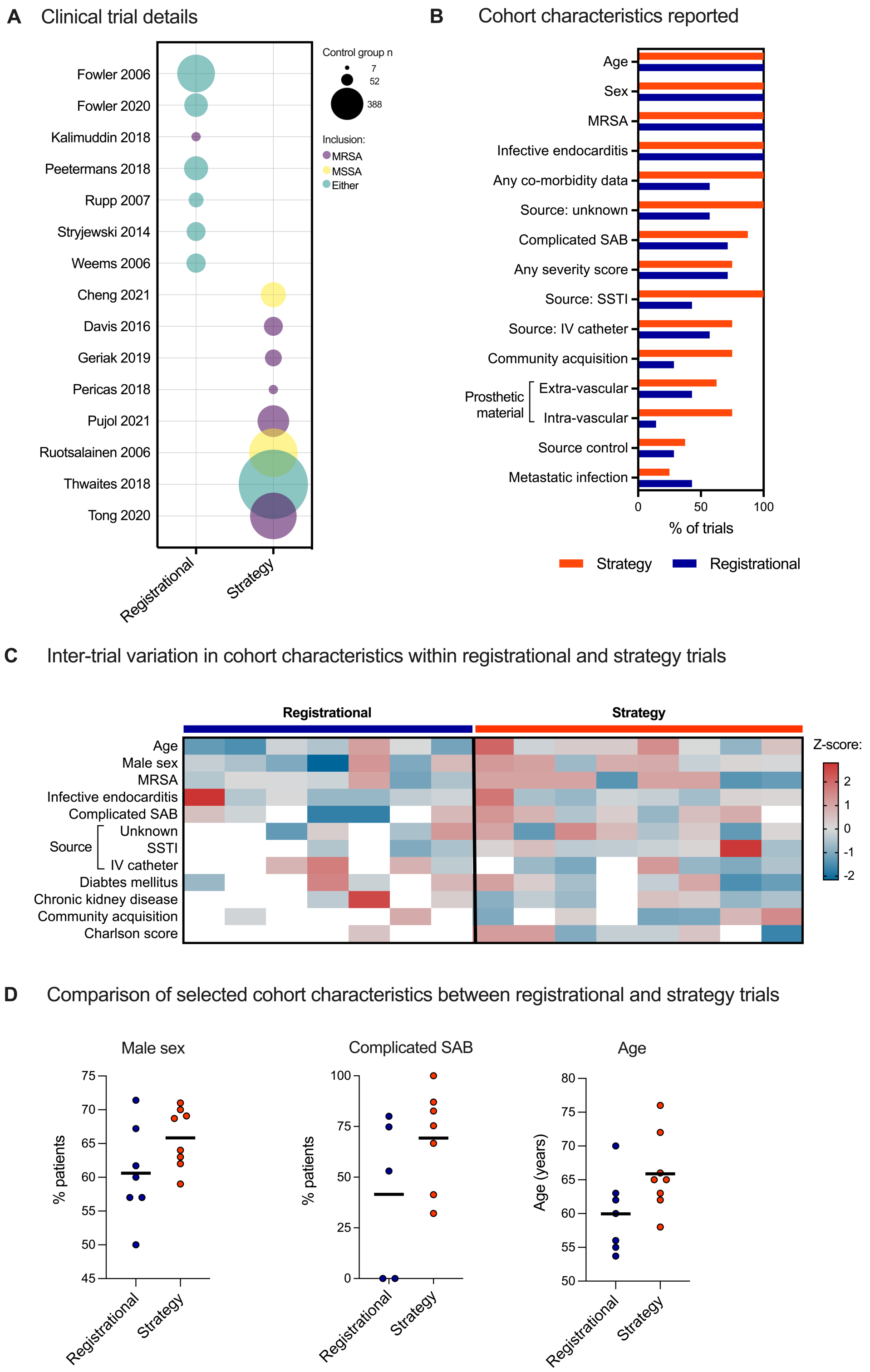

Supplementary Figure 3: Comparison of registrational and strategy trials
(A) Details of SAB RCTs stratified by trial purpose. The size of the bubble is proportional to the number of participants in the control arm. Bubbles are coloured according to the inclusion of MSSA, MRSA or both. **(B)** Cohort characteristics reported in included trials, stratified by trial purpose. **(C)** Variability in cohort characteristics between trials. Cells in the heatmap are shaded by z-score. Only variables reported in >50% of all trials were included. Blank cells represent missing values. **(D)** Comparison of selected cohort characteristics between registrational and strategy trials. Each data point represents one study. The line shows the mean (data normally distributed).

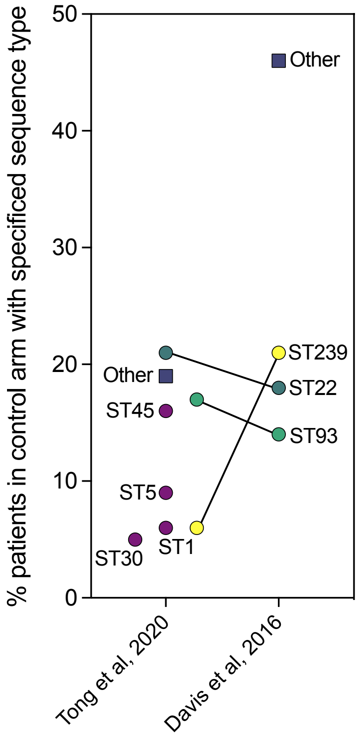

Supplementary Figure 4: Variation in *S. aureus* sequence types between trials

Two trials reported MLST data for *S. aureus* isolates; both trials recruited specifically MRSA bacteraemia. One trial was conducted in Australian hospitals[6] and the other was conducted in Australia, Singapore, Israel and New Zealand[12]. Data points in the figure represent MRSA sequence types. Lines connect data for the same sequence types reported by both trials.

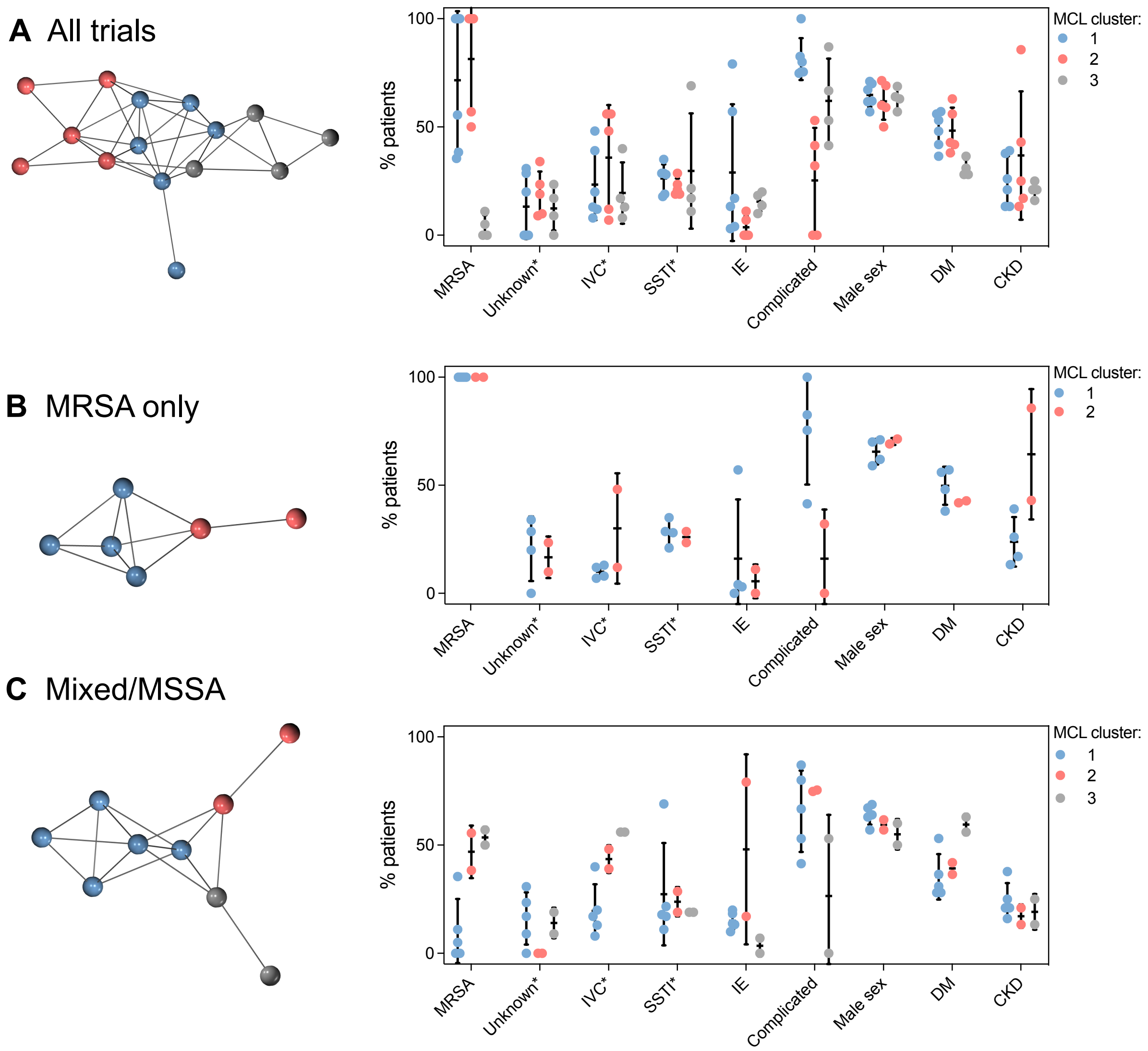

Supplementary Figure 5: Network analysis of SAB RCT cohort characteristics

Cohort characteristics were used to identify clusters of trials with similar patients included. Nodes in the network diagrams represent individual trials and are coloured by cluster membership, determined using the Markov Clustering Algorithm (a granularity of 2.6 was applied to identify >1 cluster per network). Edges represent connections with a Pearson correlation value of ≥0.62 (chosen to keep all trials in a single network). Inset dot plots show the distribution of cohort characteristics across the clusters identified in the corresponding network analysis. Lines show mean and standard deviation. Each data point represents one trial.

IVC: IV catheter; SSTI: skin and soft tissue infection; IE: infective endocarditis; DM: diabetes mellitus; CKD: chronic kidney disease.

* denotes source of SAB.

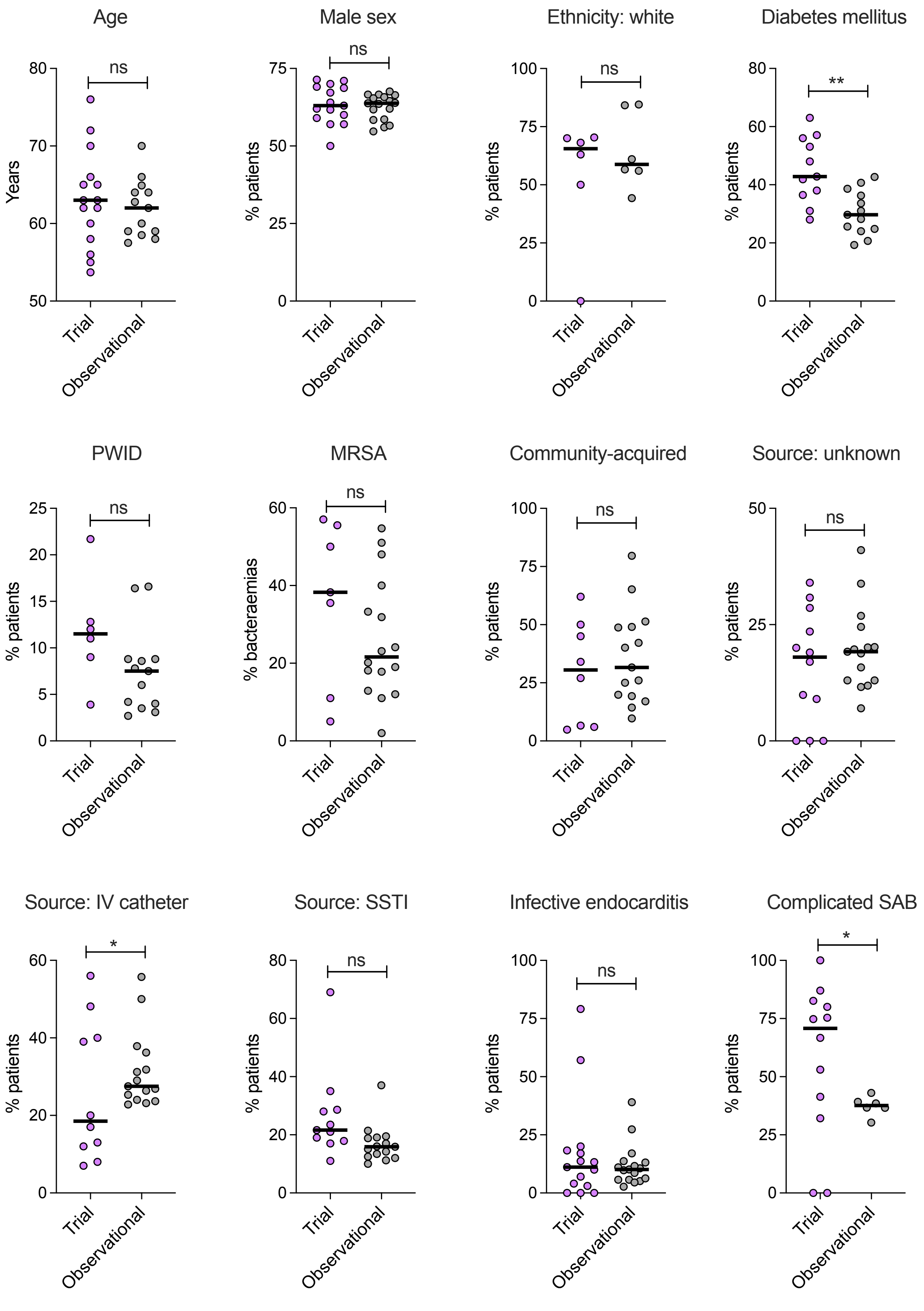

Supplementary Figure 6: SAB RCT and observational study cohort characteristics
Each data point represents one study. The line shows the median. Results of univariable analyses are shown, using unpaired t-tests (parametric data) or Mann-Whitney tests (non-parametric). Trials restricted to MRSA or MSSA only were excluded from the MRSA analysis shown here.
* p<0.05; ** p<0.01; ns: not significant.
PWID: person who injects drugs; IV: intravenous; SSTI: skin and soft tissue infection.

Supplementary references

1. Fowler VG, Jr., Boucher HW, Corey GR, et al. Daptomycin versus standard therapy for bacteremia and endocarditis caused by Staphylococcus aureus. N Engl J Med **2006**; 355(7): 653-65.
2. Ruotsalainen E, Järvinen A, Koivula I, et al. Levofloxacin does not decrease mortality in Staphylococcus aureus bacteraemia when added to the standard treatment: a prospective and randomized clinical trial of 381 patients. J Intern Med **2006**; 259(2): 179-90.
3. Weems JJ, Jr., Steinberg JP, Filler S, et al. Phase II, randomized, double-blind, multicenter study comparing the safety and pharmacokinetics of tefibazumab to placebo for treatment of Staphylococcus aureus bacteremia. Antimicrob Agents Chemother **2006**; 50(8): 2751-5.
4. Rupp ME, Holley HP, Jr., Lutz J, et al. Phase II, randomized, multicenter, double-blind, placebo-controlled trial of a polyclonal anti-Staphylococcus aureus capsular polysaccharide immune globulin in treatment of Staphylococcus aureus bacteremia. Antimicrob Agents Chemother **2007**; 51(12): 4249-54.
5. Stryjewski ME, Lentnek A, O'Riordan W, et al. A randomized Phase 2 trial of telavancin versus standard therapy in patients with uncomplicated Staphylococcus aureus bacteremia: the ASSURE study. BMC Infect Dis **2014**; 14: 289.
6. Davis JS, Sud A, O'Sullivan MVN, et al. Combination of Vancomycin and β-Lactam Therapy for Methicillin-Resistant Staphylococcus aureus Bacteremia: A Pilot Multicenter Randomized Controlled Trial. Clin Infect Dis **2016**; 62(2): 173-80.
7. Kalimuddin S, Chan YFZ, Phillips R, et al. A randomized phase 2B trial of vancomycin versus daptomycin for the treatment of methicillin-resistant Staphylococcus aureus bacteremia due to isolates with high vancomycin minimum inhibitory concentrations - results of a prematurely terminated study. Trials **2018**; 19(1): 305.
8. Pericàs JM, Moreno A, Almela M, et al. Efficacy and safety of fosfomycin plus imipenem versus vancomycin for complicated bacteraemia and endocarditis due to methicillin-resistant Staphylococcus aureus: a randomized clinical trial. Clin Microbiol Infect **2018**; 24(6): 673-6.
9. Thwaites GE, Scarborough M, Szubert A, et al. Adjunctive rifampicin for Staphylococcus aureus bacteraemia (ARREST): a multicentre, randomised, double-blind, placebo-controlled trial. Lancet **2018**; 391(10121): 668-78.
10. Peetermans M, Liesenborghs L, Peerlinck K, et al. Targeting Coagulase Activity in Staphylococcus aureus Bacteraemia: A Randomized Controlled Single-Centre Trial of Staphylothrombin Inhibition. Thromb Haemost **2018**; 118(5): 818-29.
11. Geriak M, Haddad F, Rizvi K, et al. Clinical Data on Daptomycin plus Ceftaroline versus Standard of Care Monotherapy in the Treatment of Methicillin-Resistant Staphylococcus aureus Bacteremia. Antimicrob Agents Chemother **2019**; 63(5).
12. Tong SYC, Lye DC, Yahav D, et al. Effect of Vancomycin or Daptomycin With vs Without an Antistaphylococcal β-Lactam on Mortality, Bacteremia, Relapse, or Treatment Failure in Patients With MRSA Bacteremia: A Randomized Clinical Trial. Jama **2020**; 323(6): 527-37.
13. Fowler VG, Jr., Das AF, Lipka-Diamond J, et al. Exebacase for patients with Staphylococcus aureus bloodstream infection and endocarditis. J Clin Invest **2020**; 130(7): 3750-60.
14. Pujol M, Miró JM, Shaw E, et al. Daptomycin Plus Fosfomycin Versus Daptomycin Alone for Methicillin-resistant Staphylococcus aureus Bacteremia and Endocarditis: A Randomized Clinical Trial. Clin Infect Dis **2021**; 72(9): 1517-25.
15. Cheng MP, Lawandi A, Butler-Laporte G, De l'Étoile-Morel S, Paquette K, Lee TC. Adjunctive Daptomycin in the Treatment of Methicillin-susceptible Staphylococcus aureus Bacteremia: A Randomized, Controlled Trial. Clin Infect Dis **2021**; 72(9): e196-e203.
16. Fowler VG, Jr., Olsen MK, Corey GR, et al. Clinical identifiers of complicated Staphylococcus aureus bacteremia. Arch Intern Med **2003**; 163(17): 2066-72.

17. Kaasch AJ, Barlow G, Edgeworth JD, et al. Staphylococcus aureus bloodstream infection: a pooled analysis of five prospective, observational studies. J Infect **2014**; 68(3): 242-51.
18. Kaech C, Elzi L, Sendi P, et al. Course and outcome of Staphylococcus aureus bacteraemia: a retrospective analysis of 308 episodes in a Swiss tertiary-care centre. Clin Microbiol Infect **2006**; 12(4): 345-52.
19. Laupland KB, Ross T, Gregson DB. Staphylococcus aureus bloodstream infections: risk factors, outcomes, and the influence of methicillin resistance in Calgary, Canada, 2000-2006. J Infect Dis **2008**; 198(3): 336-43.
20. Le Moing V, Alla F, Doco-Lecompte T, et al. Staphylococcus aureus Bloodstream Infection and Endocarditis--A Prospective Cohort Study. PLoS One **2015**; 10(5): e0127385.
21. Turnidge JD, Kotsanas D, Munckhof W, et al. Staphylococcus aureus bacteraemia: a major cause of mortality in Australia and New Zealand. Med J Aust **2009**; 191(7): 368-73.
22. Morris AK, Russell CD. Enhanced surveillance of Staphylococcus aureus bacteraemia to identify targets for infection prevention. J Hosp Infect **2016**; 93(2): 169-74.
23. Tong SY, van Hal SJ, Einsiedel L, Currie BJ, Turnidge JD. Impact of ethnicity and socio-economic status on Staphylococcus aureus bacteremia incidence and mortality: a heavy burden in Indigenous Australians. BMC Infect Dis **2012**; 12: 249.
24. Jenkins TC, Price CS, Sabel AL, Mehler PS, Burman WJ. Impact of routine infectious diseases service consultation on the evaluation, management, and outcomes of Staphylococcus aureus bacteremia. Clin Infect Dis **2008**; 46(7): 1000-8.
25. Robinson JO, Pozzi-Langhi S, Phillips M, et al. Formal infectious diseases consultation is associated with decreased mortality in Staphylococcus aureus bacteraemia. Eur J Clin Microbiol Infect Dis **2012**; 31(9): 2421-8.
26. Fowler VG, Jr., Sanders LL, Sexton DJ, et al. Outcome of Staphylococcus aureus bacteremia according to compliance with recommendations of infectious diseases specialists: experience with 244 patients. Clin Infect Dis **1998**; 27(3): 478-86.
27. Khatib R, Johnson LB, Fakih MG, et al. Persistence in Staphylococcus aureus bacteremia: incidence, characteristics of patients and outcome. Scand J Infect Dis **2006**; 38(1): 7-14.
28. Willekens R, Puig-Asensio M, Suanzes P, et al. "Mortality in Staphylococcus aureus bacteraemia remains high despite adherence to quality indicators: secondary analysis of a prospective cohort study". J Infect **2021**.
29. Souli M, Ruffin F, Choi SH, et al. Changing Characteristics of Staphylococcus aureus Bacteremia: Results From a 21-Year, Prospective, Longitudinal Study. Clin Infect Dis **2019**; 69(11): 1868-77.
30. Van Spall HG, Toren A, Kiss A, Fowler RA. Eligibility criteria of randomized controlled trials published in high-impact general medical journals: a systematic sampling review. Jama **2007**; 297(11): 1233-40.
31. Marks LR, Liang SY, Muthulingam D, et al. Evaluation of Partial Oral Antibiotic Treatment for Persons Who Inject Drugs and Are Hospitalized With Invasive Infections. Clin Infect Dis **2020**; 71(10): e650-e6.
